# Plasma fatty acids as diagnostic markers in autistic patients from Saudi Arabia

**DOI:** 10.1186/1476-511X-10-62

**Published:** 2011-04-21

**Authors:** Afaf K El-Ansary, Abir G Ben Bacha, Layla Y Al- Ayahdi

**Affiliations:** 1 Biochemistry Department, Science College, King Saud University, P.O Box 22452, Zip code 11495, Riyadh, Saudi Arabia; 2 Autism Research and Treatment Center, King Saud University, P O Box 2925 Riyadh 11461 Saudi Arabia; 3 Shaik AL-Amodi Autism Research Chair, King Saud University, P O Box 2925 Riyadh 11461 Saudi Arabia; 4 Medicinal Chemistry Department, National Research Centre, P O Box 12622, Dokki, Cairo, Egypt; 5 Department of Physiology, Faculty of Medicine, King Saud University, P O Box 2925 Riyadh 11461 Saudi Arabia

**Keywords:** Autism, Fatty acids, Oxidative stress, Valeric acid, Propionic acid, Polyunsaturated fatty acids

## Abstract

**Backgrounds:**

Autism is a family of developmental disorders of unknown origin. The disorder is characterized by behavioral, developmental, neuropathological and sensory abnormalities, and is usually diagnosed between the ages of 2 and 10 with peak prevalence rates observed in children aged 5-8 years. Recently, there has been heightened interest in the role of plasma free fatty acids (FA) in the pathology of neurological disorders. The aim of this study is to compare plasma fatty acid profiles of Saudi autistic patients with those of age-matching control subjects in an attempt to clarify the role of FA in the etiology of autism.

**Methods:**

26 autistic patients together with 26-age-matching controls were enrolled in the present study. Methyl esters of FA were extracted with hexane, and the fatty acid composition of the extract was analyzed on a gas chromatography.

**Results:**

The obtained data proved that fatty acids are altered in the plasma of autistic patients, specifically showing an increase in most of the saturated fatty acids except for propionic acid, and a decrease in most of polyunsaturated fatty acids. The altered fatty acid profile was discussed in relation to oxidative stress, mitochondrial dysfunction and the high lead (Pb) concentration previously reported in Saudi autistic patients. Statistical analysis of the obtained data shows that most of the measured fatty acids were significantly different in autistic patients compared to age -matching controls.

**Conclusions:**

Receiver Operating Characteristic (ROC) curve analysis shows satisfactory values of area under the curve (AUC) which could reflect the high degree of specificity and sensitivity of the altered fatty acids as biomarkers in autistic patients from Saudi Arabia.

## Introduction

Autism is a developmental disorder characterized by impaired communication and social behavioral features, as well as displays of stereotypical behavior, present in the first 3 years of life [1]. Diagnosis of autism is behavior based, and a single behavior or group of behaviors is able to distinguish autism from other developmental disorders [2]. The pathogenesis of autism is uncertain, but is thought to involve an interaction between multiple susceptibility genes and/or epigenetic effects and/or environmental factors [3-5].

Lipids are heterogeneous molecules that serve many roles, from providing cell structure to energy storage for cell signaling. The brain is one of the most lipid-enriched tissues in the human body. Infants' brains are small and undeveloped at birth and must incorporate fatty acids and cholesterol from circulation to develop properly [6]. Docosahexanoic acid (DHA) in particular is found in high abundance in the phospho- lipids of the brain contributing to membrane structure and function, eicosanoid signaling, and gene expression modulation [7,8]. DHA also plays a role in inhibition of neuronal apoptosis and in regulating neuronal excitability through GABA receptors [9,10]. Furthermore, there is evidence that developing brains obtain fatty acids transported through the blood, especially DHA [11]. Thus, examining the concentrations and compositions of plasma fatty acids may prove to be diagnostically important.

Mitochondrial fatty acid oxidation (FAO) deficiencies usually present in the neonate or toddler with hypoketotic hypoglycaemia, metabolic acidosis, mitochondrial dysfunction, hyperammonemia, muscle weakness, cardiomyopathy, seizures, psychomotor delay, developmental regression, behavioral disorders and attention deficit disorder [12-15]. Neonatal presentations are usually severe with poor prognosis and include cardiac arrhythmia and sudden death [16], however in mild phenotypes there may be an initial period of normal development and function before decompensation in association with metabolic stress or immune activation, such as fasting, illness or vaccination [17]. With the exception of cardiac involvement and sudden death, all of the metabolic and developmental abnormalities listed above may occur in autism, and the onset of autism may also be regressive following a period of initially normal infant development. This information initiates our interest to examine the plasma fatty acid profiles in autistic patients compared to age-matched control. This may explain their diagnostic and mechanistic roles in a developmental disorder such as autism.

## Material and methods

### Subjects

The study protocol followed the ethical guidelines of the most recent Declaration of Helsinki (Edinburgh, 2000). All subjects enrolled in the study (26 autistic patients and 26 age-matched controls) had written informed consent provided by their parents and assented to participate if developmentally able. They were enrolled through the ART Center (Autism Research & Treatment Center) clinic. The ART Center clinic sample population consisted of children diagnosed on the autism spectrum (ASD). The diagnosis of ASD was confirmed in all subjects using the Autism Diagnostic Interview-Revised (ADI-R) and the Autism Diagnostic Observation Schedule (ADOS) and 3DI (Developmental, dimensional diagnostic interview). The ages of all autistic children who participated were between the ages of 4 and 12 years old. All were simplex cases. All are negative for fragile x gene study. The control group recruited from well baby clinic at king Khaled university hospital with mean age 4-11 years old. Subjects were excluded from the investigation if they had organic aciduria, dysmorphic features, or diagnosis of Fragile X or other serious neurological (e.g., seizures), psychiatric (e.g., bipolar disorder) or known medical conditions. All participants were screened via parental interview for current and past physical illness. Children with known endocrine, cardiovascular, pulmonary, liver, kidney or other medical disease were excluded from the study. None of the recruited autistic patients were on special diets or alternative treatments.

### Samples collection

Blood samples were collected in the morning following at least 10 hour period of fasting. Plasma was collected using standard clinical practices and stored at -80°C until thawed for analysis.

### Fatty acid profiling

Plasma (200 μl) lipids were extracted in the presence of internal standards and FA methylated using 3N methanolic HCL in sealed vials under nitrogen and incubated at 100C for 45 min. The methyl esters of free fatty acids were extracted with hexane, and the fatty acid composition of the extract was analyzed on a gas chromatograph (Helwlett- Packard 5890 series II plus, HP analytical Direct, Wilmington, DE), equipped with a flame ionization detector and a 30 m × 0.25 mm × 0.25 μm capillary column(Omegawax 250# 2-4136, Supelco). The helium gas flow rate was 1.2 ml/min, with a split/flow ratio of 50:1. Oven temperature was held at 205°C. The injector and detector temperatures were 260 and 262°C, respectively. Two internal standards, C15:0 and C23:0, were added during analysis. Fatty acids were identified via comparison of retention times with authentic standards [18].

### Statistical analysis

An SPSS computer program was used. Results were expressed as mean ± S.D. and all statistical comparisons were made by means of independent t-Test with P ≤ 0.001 was considered significant. Reciever Operating Characteristics analysis (ROC) was performed. Area under the curve, cutoff values together with degree of specificity and sensitivity were calculated. ROC curves are constructed by plotting the false positive rate (i.e. 100-specificity) against the true positive rate (i.e. sensitivity). These have been widely accepted as standard tools for evaluating the performance of diagnostic tests. The AUC is an overall summary of diagnostic accuracy, incorporating both components of accuracy, i.e., sensitivity and specificity, into a single measure. The AUC has been widely used as a quantitative index of the performance of a biomarker in a variety of applied fields; it is a simple and convenient overall measure of diagnostic test [19,20].

## Results

**Figure 1:**
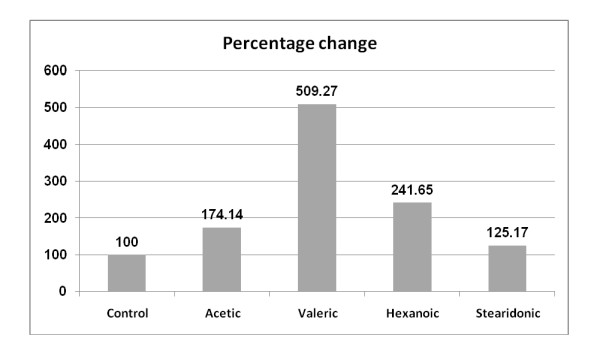
**Percentage change in valeric, hexanoic and stearidonic acids of autistic patients (N = 26) compared to control (N = 26)**.

**Figure 2:**
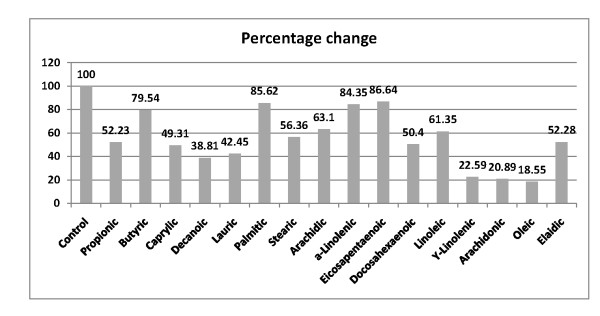
**Percentage decrease of saturated and unsaturated fatty acids in plasma of autistic patients (N = 26) compared to control (N = 26), presented as the first bar to the left showing 100% value**.

Results are presented in tables 1, 2, 3 &4 and figures 1, 2, &3. Fatty acids concentrations were calculated as mmoles/L plasma. Values are expressed as mean ± S.D, P value between all controls and all autistic subjects is reported for each fatty acid and illustrated as a star when P < 0.001.

**Table 1:** Mean ± S.D of plasma levels of acetic, valeric, hexanoic and stearidonic saturated fatty acids in autistic patients compared to age- matching controls

Fatty acid	Group	N	**Mean ± S.D**.	Percentage change	P value
Acetic	Control	26	0.558 ± 0.082	100.00	0.000
		
	Autistic	26	0.972 ± 0.247	174.14	

Valeric	Control	26	0.100 ± 0.015	100.00	0.000
		
	Autistic	26	0.510 ± 0.229	509.27	

Hexanoic	Control	26	0.597 ± 0.478	100.00	0.000
		
	Autistic	26	1.442 ± 0.349	241.65	

Stearidonic	Control	26	0.363 ± 0.122	100.00	0.009
		
	Autistic	26	0.455 ± 0.120	125.17	

This table describes the independent t-test between the control and autistic groups regarding levels of acetic, valeric, hexanoic and stearidonic acids expressed in mmol/L plasma. Significant level at p < 0.001

**Table 2:** Mean ± S.D of saturated, mono and polyunsaturated fatty acids in plasma of autistic patients compared to controls

Fatty acid	Group	N	**Mean ± S.D**.	Percentage change	P value
Propionic	Control	26	1.674 ± 0.441	100.00	0.000
		
	Autistic	26	0.874 ± 0.249	52.23	

Butyric	Control	26	0.738 ± 0.211	100.00	0.028
		
	Autistic	26	0.587 ± 0.267	79.54	

Caprylic	Control	26	2.250 ± 0.481	100.00	0.000
		
	Autistic	26	1.109 ± 0.281	49.31	

Decanoic	Control	26	1.954 ± 0.750	100.00	0.000
		
	Autistic	26	0.759 ± 0.182	38.81	

Lauric	Control	26	1.903 ± 0.574	100.00	0.000
		
	Autistic	26	0.808 ± 0.160	42.45	

Palmitic	Control	26	1.905 ± 0.537	100.00	0.037
		
	Autistic	26	1.631 ± 0.372	85.62	

Stearic	Control	26	1.219 ± 0.315	100.00	0.000
		
	Autistic	26	0.687 ± 0.281	56.36	

Arachidic	Control	26	0.673 ± 0.174	100.00	0.000
		
	Autistic	26	0.425 ± 0.222	63.10	

α-Linolenic	Control	26	0.354 ± 0.119	100.00	0.045
		
	Autistic	26	0.299 ± 0.067	84.35	

Eicosapentaenoic	Control	26	0.328 ± 0.112	100.00	0.245
		
	Autistic	22	0.284 ± 0.145	86.64	

Docosahexaenoic	Control	26	0.754 ± 0.340	100.00	0.000
		
	Autistic	22	0.380 ± 0.097	50.40	

Linoleic	Control	26	0.359 ± 0.162	100.00	0.023
		
	Autistic	26	0.220 ± 0.255	61.35	

γ-Linolenic	Control	26	0.716 ± 0.323	100.00	0.000
		
	Autistic	20	0.162 ± 0.101	22.59	

Arachidonic	Control	26	0.574 ± 0.202	100.00	0.000
		
	Autistic	20	0.120 ± 0.040	20.89	

Oleic	Control	26	1.212 ± 0.518	100.00	0.000
		
	Autistic	26	0.225 ± 0.064	18.55	

Elaidic	Control	26	0.234 ± 0.080	100.00	0.000
		
	Autistic	26	0.122 ± 0.099	52.28	

This table describes the independent t-test between the control and autistic groups specified saturated, mono and polyunsaturated fatty acids. Fatty acids are expressed in mmol/L plasma. Significant level at p < 0.001

**Table 3:** Roc analysis - related data of valeric, hexanoic and stearidonic saturated fatty acids

Fatty acid	Area under the curve	Best Cutoff value	Sensitivity %	Specificity %
Acetic	0.985	0.684	92.3%	92.3%

Valeric	1.000	0.196	100.0%	100.0%

Hexanoic	0.916	1.009	92.3%	88.5%

Stearidonic	0.734	0.344	80.8%	65.4%

**Table 4:** Roc analysis - related data of specified saturated, mono and polyunsaturated fatty acids

Fatty acid	Area under the curve	Best Cutoff value	Sensitivity %	Specificity %
Propionic	0.963	1.279	92.3%	84.6%

Butyric	0.655	0.689	65.4%	50.0%

Caprylic	0.972	1.617	100.0%	92.3%

Decanoic	0.996	1.197	100.0%	92.3%

Lauric	1.000	1.214	100.0%	100.0%

Palmitic	0.631	1.874	69.2%	46.2%

Stearic	0.897	0.975	84.6%	84.6%

Arachidic	0.823	0.462	76.9%	92.3%

a-Linolenic	0.611	0.325	69.2%	50.0%

Eicosapentaenoic	0.617	0.283	54.5%	57.7%

Docosahexaenoic	0.876	0.427	77.3%	84.6%

Linoleic	0.791	0.162	76.9%	100.0%

Y-Linolenic	0.996	0.338	100.0%	96.2%

Arachidonic	1.000	0.271	100.0%	100.0%

Oleic	1.000	0.463	100.0%	100.0%

Elaidic	0.831	0.213	80.8%	76.9%

**Figure 3:**
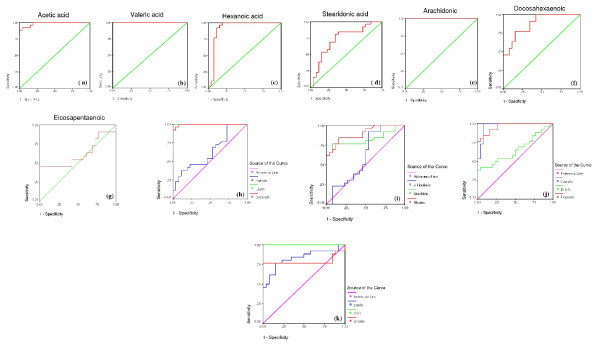
**Roc curves demonstrate AUC, specificity and sensitivity values of different fatty acids as biomarkers of autism**.

Table 1 and figure 1 demonstrate the elevated plasma levels in autistic patients of the following saturated fatty acids: acetic, valeric, hexanoic and stearidonic. While stearidonic acid shows the least percentage increase (25%), valeric acid shows highest plasma levels in autistic patients compared to controls (409.27%). Acetic and hexanoic acids gave 74.14 and 41.65% increase respectively.

On the other hand, Table 2 demonstrates the reduced levels of different saturated, mono or polyunsaturated fatty acids in autistic patients when compared to control subjects. Figure 2 shows different percentage decrease of saturated (propionic, butyric, caprylic, decanoic, lauric, palmitic and stearic) together with mono (oleic) and polyunsaturated fatty acids. Oleic, arachidonic, γ-linolenic, decanoic, caprilic and propionic were the most significantly impaired levels recorded percentage decreases of 81.45, 79.11, 77.41, 61.14, 50.6 and 47.77%, respectively.

ROC curves are presented in figure 3 (a-k). Area under the curve, cutoff values together with degree of specificity and sensitivity are presented in table 3 and 4 respectively. It could be easily noticed that autistic patients have remarkably different fatty acid profiles compared to controls. Most of the measured fatty acids recorded high values of specificity and sensitivity.

## Discussion

Biomarkers should ideally be quantitative biological measures with an accurate indication of a specific mechanism and ideally are not invasive. Identifying biomarkers will almost certainly lead to a better understanding of the pathogenesis required to design the most effective treatments of autism.

The present study recorded high significant alterations in the fatty acid profiles of autistic patients compared to age-matching controls. Table 1 demonstrates levels of certain saturated fatty acids in both groups. It could be easily seen that short chain fatty acids represented by acetic, valeric, hexanoic and stearidonic acids were significantly higher in autistic patients compared to control (table 1 and figure 1). The significantly higher level of acetic acid could be easily related to the gastrointestinal inflammation as one of the most common clinical presentation of autism. It is well known that acetic acid induced colitis is a well -established model [21-23] whereby acetate ions cause massive intracellular acidification resulting in injury of epithelial cells and inflammatory response [24].

The most remarkable elevation in fatty acid level in the present study was observed in valeric acid. The extremely high level of valeric acid ( 409.27% increase) could be related to the increase of α-keto β-methyl valeric acid (KMV) derivative previously reported as an antioxidant highly effective in neutralizing H_2_O_2 _oxidative stress [25]. The authors reported that tissue accumulation and high urinary excretion of KMV occurred in maple syrup urine disease (MSUD) which shows neurological dysfunction. They assessed the effect of KMV on the activities of the mitochondrial respiratory chain complexes in cerebral cortex from young rats in an attempt to elucidate the biochemical defect responsible for the inhibition of aerobic metabolism (lower CO_2 _formation) and activation of anaerobic glycolysis (increased lactate release). They recorded that KMV inhibited complex I-III of the respiratory chain and significantly stimulated lactate production by around 20-30%. Therefore, it may be concluded that KMV could alter the energy metabolism in brain cortex. In addition, the high level of plasma valeric acid observed in the present study could be easily explained and correlated to the H_2_O_2 _oxidative stress, impaired energy metabolism, and elevated plasma lactate previously recorded in Saudi autistic patients [26-29].

The recent work performed by MacFabe et al. (2007) [30] concerns the role of a panel of gut-borne factors in autism spectrum disorder through using a novel rodent model. The transport of propionate and butyrate across the blood brain barrier was previously ascertained [31-34]. These authors demonstrated that intracerebroventricular infusions of short chain fatty acids produced behavioral (hyperactive, preservative, social impairments), electrophysiological (seizure, caudate spiking), biochemical (increased oxidative stress, reduced glutathione, lipids) and neuropathological (innate neuroinflammation) changes in adult rats consistent with those seen in autism. References regarding the further development of this model from the behavioral to biochemical levels will further support this study findings and reduce the plausible argument that the lipid alterations are merely an epiphenomena caused by a poor diet and have nothing to do with autism pathophysiology per se. ^14^C propionate and ^14^C hydroxybutyrate were found to cross the blood-brain barrier with brain uptake indexes of 43.53 and 7.10%, respectively. Transport of both of these substrates was saturable, with the values of transport Km being 2.03 and 6.54 mM, respectively. These differences in brain uptake indexes and transport Km values could be used to explain the obtained lower plasma propionate than butyrate recorded in the present study in autistic patients compared to healthy age-matching controls. Since the lower the Km, the higher the affinity of the transporters for the substrates, so an uptake index of 43.53% and a km value of 2.03 are enough to facilitate the penetration of propionate to the brain cell.

The observed results could confirm the previous work of MacFabe et al. (2007) [30] concerning the role of PPA in the etiopathology of autism and the possibility to use this short chain fatty acid to induce autism like behaviors and biochemical changes in animal model. Lower level of PPA in plasma of autistic patients could reflect the remarkable higher rate of uptake with brain.

There is growing interest in the roles of n-3 PUFA docosahexaenoic acid (DHA) and precursor eicosapentaenoic acid (EPA) in brain structure, function and mental health [35-37]. The longest chain n-3 DHA is the most abundant PUFA in brain membrane phospholipids indicative of its role in membrane fluidity and associated metabolic and neural activities. DHA is particularly concentrated at neural synapses, sites of neurotransmitter signalling. Omega-6 PUFA AA is also abundant in the brain reflecting a key role for brain structure and function. AA precursor, gamma-linolenic acid (GLA), and n-3 DHA precursor EPA are thought to be important for brain function via eicosanoid synthesis. EPA may be particularly important for production of eicosanoids with anti-inflammatory, anti-thrombotic, and vasodilatory properties [37].

PUFA have been proclaimed as critical for intellectual growth and development in the developing neonatal/infant brain and in early childhood [38]. Given that brain development, particularly executive functioning (EF), continues throughout childhood [39,40], PUFA could also play an important role in cognitive function in older children. In addition, PUFA have been specifically associated with dopamine activity in the frontal lobes of the brain [41], which may impact directly on EF, and has been associated with Attention Deficit Hyperactivity Disorder (ADHD) [42].

Infants' brains are small and undeveloped at birth and must incorporate fatty acids and cholesterol from circulation to develop properly [43]. DHA plays a role in inhibition of neuronal apoptosis and in regulating neuronal excitability through GABA receptors [9,10]. Furthermore, there is evidence that developing brains obtain fatty acids transported through the blood, especially DHA [11].

The present investigation also indicated significantly lower levels of most PUFA (table 2 and figure 2), with exception of EPA which recorded non-significant lower concentration. This is in good agreement with the previous reports of Vancassel et al (2001) [44] who recorded decreased levels of the essential fatty acid DHA with normal levels of its omega-3 precursor in autism. Decreased DHA together with mild to moderate increases in lactate were observed in long chain FAO disorders [45]. Based on these observations, the lower level of DHA observed in the present study could be easily correlated to the previously reported increase of lactic acid in Saudi autistic patients [46].

In addition, lower level of AA recorded in the present work could find support through considering the work of Wiest et al. (2009) who attributed the impaired inflammatory responses seen in autistic patients to the diminished AA level in their plasma [47].

Diets rich in saturated fats may increase brain uptake of intact free fatty acids from the plasma through the blood brain barrier (BBB) [48], the BBB is not a barrier for fatty acids [49]. Patil and Chan (2005) study the role of FFAs in causing hyperphosphorylation of tau in primary rat cortical neurons [50]. The observed FFA-astrocyte-induced hyperphosphorylation of tau was reduced by co-treatment of neurons with 10mM *N*-acetyl cysteine (NAC), an antioxidant suggesting a central role of astroglia-mediated oxidative stress in the FFA-induced hyperphosphorylation of tau in neurons. Several studies have shown a relationship between elevated tissue Pb and oxidative stress biomarkers and fatty acid composition [51-54]. The impaired fatty acid profile recorded in the present study could be easily supporting the previous reports which demonstrate high plasma Pb, elevated lipid peroxidation and low GSH levels in Saudi autistic patients compared to control [46].

It is proposed that saturated fatty acids (SFA) induce BBB dysfunction and delivery of toxic substances from blood- to-brain [55]. Moreover, Morgan (2009) [56] suggests that the underlying toxicity of SFA is a consequence of disturbances in protein processing and endoplasmic reticulum dysfunction, for example apoptotic induction. The present recognition of short chain fatty acids as biomarkers in Saudi autistic children could be supported through considering lipid-related biomarkers recorded in other neurological diseases. Miyake et al (2010) [54] observed positive relationship between cholesterol intake and the risk of Parkinson's disease (PD) but he recorded unaltered SFA levels in PD patients which is incompatible with the hypercholesterolemic effect of these acids. In addition, an emerging body of evidence is consistent with the hypothesis that dietary fats influence Alzheimer's disease (AD) risk, but less clear is the mechanisms by which this occurs. Accumulating evidence that dietary fats, specifically, chronic ingestion of saturated fats significantly influence cerebrovascular integrity and as a consequence altered amyloid beta (Aβ) kinetics across the BBB [55]. Regarding the role of saturated fatty acids in the etiology of autism, impaired mitochondrial FAO, elevated plasma very long chain saturated fatty acids (VLCFAs) was recorded as putative causal factors in the biochemistry, neuropathology, and gender bias in autism. This study is the first study which highlights the contribution of short chain fatty acids in the etiology of autism.

ROC curves are constructed by plotting the false positive rate (i.e. 100-specificity) against the true positive rate (i.e. sensitivity). These have been widely accepted as standard tools for evaluating the performance of diagnostic tests. The AUC is an overall summary of diagnostic accuracy, incorporating both components of accuracy, i.e., sensitivity and specificity, into a single measure. The AUC has been widely used as a quantitative index of the performance of a biomarker in a variety of applied fields; it is a simple and convenient overall measure of diagnostic test [19,20]. According to the presented ROC related data (tables 3 and 4, figure 3), it could be easily concluded that impaired fatty acid profiles could be used as diagnostic biomarkers in Saudi autistic patients. Most of the measured fatty acids show a satisfactory level of specificity and sensitivity.

## Competing interests

The authors declare that they have no competing interests.

## Authors' contributions

AE designed the study and drafted the manuscript. ABB helped to draft the manuscript and performed the statistical analysis. LA provided samples and participated in the design of the study. All authors have read and approved the final manuscript.
